# Reversed Phase-Liquid Chromatography for Recombinant AAV Genome Integrity Assessment

**DOI:** 10.1021/acs.analchem.3c00222

**Published:** 2023-05-23

**Authors:** Christoph Gstöttner, Andrei Hutanu, Sacha Boon, Aurelia Raducanu, Klaus Richter, Markus Haindl, Raphael Ruppert, Elena Domínguez-Vega

**Affiliations:** † Center for Proteomics and Metabolomics, Leiden University Medical Center, 2333ZA Leiden, The Netherlands; ‡ Pharma Technical Development Analytics, F. Hoffmann-La Roche AG, 4070 Basel, Switzerland; § Pharma Technical Operation Cell- and Gene Therapy, Roche Diagnostics GmbH, 82377 Penzberg, Germany; ∥ Coriolis Pharma Research GmbH, 82152 Planegg, Germany

## Abstract

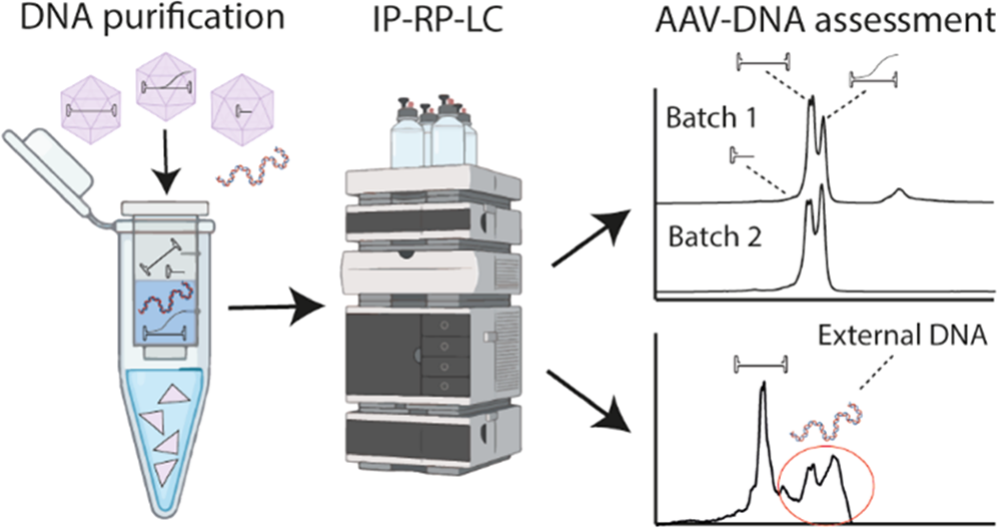

After decades of research, gene therapy products have reached market maturity in recent years. Recombinant adeno-associated viruses (rAAVs) are one of the most promising gene delivery vehicles and are currently under intense scientific investigation. These next-generation medicines remain very challenging when it comes to designing appropriate analytical techniques for quality control. One critical quality attribute is the integrity of ssDNA incorporated in these vectors. The genome is the active compound driving rAAV therapy and therefore requires proper assessment and quality control. Current techniques for rAAV genome characterization include next-generation sequencing, quantitative polymerase chain reaction, analytical ultracentrifugation (AUC), and capillary gel electrophoresis (CGE), yet each of them presents their limitations or lack of user-friendliness. In this work, we demonstrate for the first time the potential of ion pairing-reverse phase-liquid chromatography (IP-RP-LC) to characterize the integrity of rAAV genomes. The obtained results were supported by two orthogonal techniques, AUC and CGE. IP-RP-LC can be performed above DNA melting temperatures, avoiding the detection of secondary DNA isoforms, and does not require the use of dyes due to UV detection. We demonstrate that this technique is suitable for batch comparability, different rAAV serotypes (AAV2 and AAV8), internal vs external (inside vs outside the capsid) DNA analysis, and contaminated samples. Overall, it is exceptionally user-friendly, needs limited sample preparation, has high reproducibility, and permits fractionation for further peak characterization. All of these factors add significant value of IP-RP-LC to the analytical toolbox of rAAV genome assessment.

## Introduction

Gene therapy products using viral delivery vehicles, such as recombinant adenovirus, lentivirus, or adeno-associated virus (AAV), are recently gaining broad attention in the pharmaceutical industry. In particular, rAAVs combine several attributes that make them predestined for use as a gene delivery vehicle. From a safety perspective, the site-specific genome integration,^1^ lack of pathogenicity in humans,^2^ and dependency on helper viruses^3^ are very favorable. Additionally, there are multiple rAAV serotypes with different tropisms for a specific tissue permitting it to be used to treat diseases very specifically.^4^ Until 2022, already 136 clinical trials of recombinant AAV (rAAV) in different phases were reported, with two rAAV therapies for retinal dystrophy and spinal muscular atrophy authorized by the FDA.^5^ AAVs are nonenveloped viruses containing a protein capsid built of three different proteins, namely, VP1, VP2, and VP3. The wild-type capsid contains a single-stranded DNA (ssDNA) genome with a length of up to 4.8 kilobase pairs (kbp).^6^ This ssDNA usually contains two open reading frames, coding for proteins necessary for replication, capsid formation, and assembly.^7^ Both ends of the ssDNA of AAVs are flanked by inverted terminal repeats (ITRs) that form dsDNA hairpin loops. They serve as primers for DNA polymerase and thus are crucial for genome replication. Furthermore, they are necessary for the loading of the genome into the capsid.^8^ For therapeutic purposes, AAV vectors are recombinantly produced, and the aforementioned genes in between the two ITRs can be exchanged by a transgene cassette, preventing rAAV from replicating in patients.^7^ After entering the target cells and delivering ssDNA into the nucleus, double-stranded DNA (dsDNA) episomes are formed.^9,10^ These are stable and lead to a long-term expression of the gene of interest in patients allowing treatment of genetic diseases.^11−13^

In addition to the intended genome, rAAV preparations can contain several other DNA impurities, which can either be located outside the capsid and are copurified during production or inside the capsid. While the external DNA might potentially contain host cell DNA, it can also consist of plasmids used for rAAV production and, thereby, resistance cassettes that are necessary for cell selection during rAAV production.^14^ The copurified DNA is, in most of the cases, removed by a Benzonase or DNase I treatment during downstream processing.^15,16^ These species should be monitored carefully due to their potential genotoxicity.^17^ Another source of external DNA can be encapsidated DNA, which can be ejected from the capsid when samples are stressed.^18,19^ Next to the external, also internal DNA can contain selection markers but also fragments or truncated forms of the intended genome.^20−23^ All of these species are potentially immunogenic and/or genotoxic for patients.^17^ For this reason, profound characterization of the rAAV genome is of the utmost importance to ensure a safe and effective product. Another critical quality attribute (CQA) relates to the rAAV genome, which is transferred to human cells and therefore needs to be monitored carefully. Generally, only the region flanked by the two ITRs is loaded into the rAAV capsids; however, some reports describe the encapsidation of the host cell or plasmid backbone DNA.^17,21,23^ This can be especially concerning since the production plasmids contain antibiotic resistance genes and other sequences, which may lead to the expression of immunogenic proteins or peptides.^24^ These impurities range from 1 to 5%^21^ but can account for the majority (>80%) of the genome when the unfavorable rep/cap helper plasmid and transfer plasmid combinations are used.^20^ Some of them can be a product of reverse transcription of the plasmid necessary for rAAV production. This can be minimized by using plasmids that have backbones exceeding the maximum loading capacity of around 4.8 kbp of AAVs.^24^

rAAV-DNA impurities are currently characterized by quantitative real-time PCR (qPCR),^25,26^ next-generation sequencing (NGS),^27,28^ capillary gel electrophoresis (CGE),^18,29^ and analytical ultracentrifugation (AUC).^18,20^ qPCR is very sensitive and able to detect single DNA molecules. However, a disadvantage of qPCR is its biased approach relying on specific primers that can only detect certain DNA sequences and therefore is at risk of missing noncommonly observed DNA impurities.^30,31^ NGS can give a good overview of the genome integrity, with single-molecule real-time (SMRT) sequencing being the gold standard for rAAVs due to its ability to sequence entire rAAV genomes without the need for in silico reconstruction.^27,28,32^ The generation of dsDNA (necessary to ligate the SMRTbells) can be achieved either by annealing self-complementary rAAV-DNA or by extension of the 3′ end by terminal transferase. One drawback of this technique is the bias toward smaller molecules, therefore under-representing longer DNA fragments.^27^ In addition, DNA fragments that are unable to ligate to a SMRTbell adapter can be missed.^27,28^ For these reasons, complementary techniques able to directly analyze the ssDNA genome without any need for further sample manipulation are required. In contrast to the mentioned techniques, AUC analyzes intact unmodified viruses and allows the evaluation of empty and filled capsids based on their different densities and different sedimentation properties. Thus, it can be used to distinguish between full and empty capsids but also between rAAV capsids containing ssDNA with large size differences (2.1 vs 4.3 kbp).^20,33^ However, a downside of AUC is that it requires high volumes of rAAV samples.^33^ Overall, qPCR, NGS, and AUC require a high level of expertise but also long sample preparation and analysis time.^30^ CGE has demonstrated to be able to separate different DNA species found in rAAV and thereby represents a fast and economic possibility to test the genome integrity.^18,29^ Yet, no full denaturation of dsDNA to ssDNA can be achieved, so under commonly used analysis conditions, some dsDNA artifacts can form, complicating the data analysis.^34^ In addition, CGE has the benefit of very low sample consumption with injection volumes in the nanoliter range. However, for low-concentrated samples, this can be a drawback, as it limits sensitivity, especially when UV detection is employed. Moreover, the low sample volume represents a significant hurdle when it comes to fractionation, limiting further characterization options. Ion pairing reversed-phase liquid chromatography (IP-RP-LC) has, due to its high user-friendliness and robustness, also great potential to characterize DNA samples.^35^ The separation mechanism is based on the use of a lipophilic ion-pair reagent, such as triethylamine (TEA), which binds to the negatively charged DNA phosphate backbone. This ion-paring effect results in an increased hydrophobicity with an increase in DNA length, permitting the separation of differently-sized DNA species in an RP column. Another influence on the separation of different DNA species results from the different hydrophobicity of the DNA bases.^36^ Up to date, IP-RP-LC has been applied to analyze RNA samples^37,38^ as well as short and medium-sized DNA^35,39^ (up to 100 bp) fragments. Here, we demonstrate for the first time the potential of IP-RP-LC to assess the genome integrity of rAAVs with transgene sizes ranging from 2.5 to 4.6 kbp and compare our results with the two orthogonal techniques, AUC and CGE.

## Experimental Section

### Samples and Chemicals

Reagents and materials used for this study were at least of analytical grade; for more details, see Supporting Information Method S1. AAV2-V, AAV8-V, and AAV8-HEK samples were purchased from supplier 1, while AAV2-S was from supplier 2.

### Preparation of the Plasmid and rAAV-DNA for IP-RP-LC

10 or 20 μL of the rAAV sample (titers between 8 × 10^12^ and 2 × 10^13^ vg) was mixed with 50 μL of PB buffer (QIAquick PCR purification kit, Qiagen), loaded onto a QIAquick column, and centrifuged at 16,100*g* for 1 min. Afterward, the sample was washed with 750 μL of PE buffer (QIAquick PCR purification kit, Qiagen) and centrifuged for 1 min. After discarding the flow-through and centrifugation for an additional 1 min, the sample was eluted with 50 μL of elution buffer (QIAquick PCR purification kit, Qiagen). Before analysis, the sample was heat treated at 95 °C for 2 min, followed by 5 min incubation on ice. For DNA digestion, Benzonase was diluted 1:10 in a 10× digestion buffer (100 mM Tris–HCl, 25 mM MgCl_2_, 5 mM CaCl_2_ at a pH 7.6). 17 μL of the purified DNA sample was mixed with 2 μL of 10× digestion buffer and 1 μL of the diluted Benzonase. The mixture was incubated for 1 or 2 h, and digestion was stopped by heating the samples to 95 °C for 2 min, followed by 5 min cool-down on ice. For plasmid digestion, 5 μg of plasmid DNA was mixed with the provided digestion buffer as instructed by the manufacturer. 10 U of the enzyme per μg of DNA in a total volume of 100 μL was used and incubated for 60 min at 37 °C. The absence of proteins was confirmed using RP-LC (Supporting Information Method S2).

### Analysis of the Plasmid and rAAV-DNA with IP-RP-LC

For the analysis of plasmid or rAAV-DNA, an Agilent 1200 series instrument equipped with a quaternary pump (G1311A) combined with a degasser (G1322A), an autosampler (G1367D) with a thermostat (G1330B), a column oven (1316B), and a variable wavelength detector (G1314C) with a standard cell (Agilent Technologies, Waldbronn, Germany) was employed. Analysis of rAAV-DNA or the plasmid digest was performed using a DNA-PAC column at 95 °C with 0.1 M TEAA in water pH 7 (adjusted with TEA) as mobile phase A and 0.1 M TEAA in 75% H_2_O with 25% ACN pH 7 as mobile phase B. The starting condition was 35% B, which was gradually increased to 70% B in 18 min, followed by an increase to 100% B in 2 min. After 5 min of cleaning of the column at 100% B, the starting conditions were reached during a 3 min gradient, followed by re-equilibration of the column for 7 min, resulting in a total analysis time of 35 min with a constant flow rate of 0.4 mL/min. The injection volume was either 20 μL per purified ssDNA rAAV sample or 3 μL digested plasmid DNA. rAAV-ssDNA was detected using UV detection at 260 nm. The runs were aligned using a FastRuler Middle Range DNA Ladder, which was measured together at the beginning and end of each sequence.

### Preparation and Analysis of the Plasmid and rAAV-DNA for CGE-LIF

5 or 20 μL of the rAAV sample was prediluted with 1× phosphate-buffered saline (PBS) (Sigma-Aldrich) as required for the specific analysis. Afterward, samples (rAAV and digested plasmid) were purified following the instructions from the QIAquick PCR purification kit but with two washing steps of the QIAquick column. Before injection in CGE, the sample was heated at 70 °C for 2 min, followed by 5 min on ice. For analysis, a SCIEX PA800 Plus system (Brea) equipped with a solid-state laser with an excitation wavelength of 488 nm and a 520 nm bandpass emission filter (Cat. no. 65-699) from Edmund Optics (Barrington) with a temperature-controlled autosampler (±2 °C) was used. Data were acquired with 32 Karat software 10.3. Separation was performed in a bare fused silica capillary with a 50 μm internal diameter and 20 cm effective and 30 cm total length. As gel buffer 2% PVP, 4 M urea in a 1× TBE solution with 1:25,000 diluted SYBR green II was used.^29,40,41^ Voltage was set at −6 kV. The electrokinetic injection was performed for 30 or 60 s at a voltage of −5 kV. The capillary temperature was set to 25 and 10 °C was used for the autosampler.

### Preparation and Analysis of rAAV Samples Using Sedimentation Velocity Analytical Ultracentrifugation (SV-AUC)

Before analysis, the samples were thawed and equilibrated at room temperature, followed by the measurement of UV absorbance spectra (using a NanoDrop One UV–Vis spectrophotometer from Thermo Fisher Scientific) in the wavelength range of 220–350 nm to confirm a suitable initial sample concentration. Afterward, samples were diluted to an optical density (at 230 nm) of 0.8 except for AAV8 produced in HEK cells, which already had an optical density below 0.8. For measurements of sedimentation velocity, an Optima analytical ultracentrifuge from Beckman-Coulter (Brea, California) with an 8-hole AN-50 Ti analytical rotor and 12 mm charcoal epon double-sector centerpieces was used. Experimental conditions are detailed in Supporting Information Method S3.

## Results and Discussion

### Development of the IP-RP-LC Method for rAAV Genome Characterization

The genomic material in AAV-based gene therapy products is encapsidated within three structural proteins, namely, VP1, VP2, and VP3. As these proteins can influence the separation performance in IP-RP-LC by coeluting with some ssDNA species, purification of DNA from the rAAV sample is required prior to analysis. For this purpose, we performed a silica membrane-based DNA purification step using commercially available spin columns. For the approach, we use a limited amount of the rAAV sample for purification (10 or 20 μL with a titer of 2 × 10^13^ to 8 × 10^12^ vg, respectively), which resulted in 50 μL of purified DNA. This allowed duplicate analysis from a single DNA purification with 20 μL injection volume for each measurement. To ensure complete removal of the protein, the sample was analyzed before and after purification using RP-LC (Figure S1). Before DNA purification, the proteins could be detected, while no proteins were visible after DNA purification, excluding any interference during the DNA separation.

**Figure 1:**
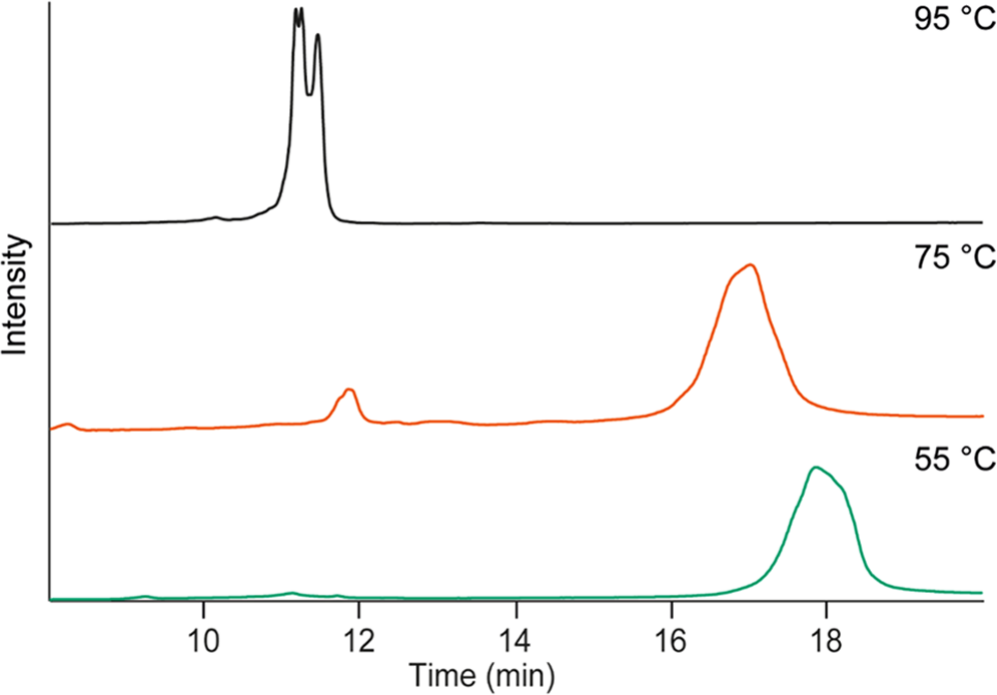
IP-RP-LC analysis of the purified AAV8-V genome at 55 °C (green trace), 75 °C (orange trace), and 95 °C (black trace).

Genomes of rAAVs are ssDNA; however, due to the nature of the hairpin-shaped ITRs, the DNA molecule can have either sense or antisense polarity.^42^ When the DNA of rAAVs is purified for analysis, dsDNA, as well as sophisticated multiplexes, may form alongside the ssDNA genome. Therefore, it is important to avoid all forms of dsDNA as these sample preparation artifacts can add complexity to the analysis. To reduce additional signals arising from these species, the use of high temperatures following a fast cooling on ice is often used.^18^ In our case, we heated the sample to 95 °C (above the melting temperature of dsDNA, around 85–90 °C depending on the sequence) for 2 min, followed by 5 min incubation on ice prior to analysis. The high temperatures should melt dsDNA into ssDNA and then lead to the formation of internal double strands in the ITR region of the rAAV genomes due to the rapid cooling and potentially limiting annealing between sense and antisense strands. However, as shown by Hutanu et al.,^18^ this approach is not fully efficient, and still, some dimers can form, resulting in additional signals. In IP-RP-LC, oligonucleotides and RNA are commonly analyzed at a temperature of 50 °C,^38,43,44^ with few reports showing that with an increased temperature up to 80 °C, resolution can be improved.^37,39^ Huang et al. showed that increasing the temperature from 50 to 65 °C simplified the number of RNA conformation structures detected.^44^ To avoid rearrangements and ensure the detection of rAAV and its impurities in the single-stranded form, we evaluate the possibility of performing the analysis above DNA melting temperatures. Figure 1 shows a comparison of an AAV8-V DNA sample analysis at 55, 75, and 95 °C. At 55 °C, a main signal between 17 and 19 min was observed. Because this temperature favors the formation of dsDNA, this signal most likely represents dsDNA species. Increasing the temperature to 75 °C resulted in two clusters of signals at 12 and 17 min, suggesting the coexistence of two populations during the separation (ssDNA and dsDNA, respectively). Since 75 °C is below the melting temperature, only a small fraction of DNA is in the single-stranded form. This is also supported by the separation mechanism of IP-RP: dsDNA pairs with a higher number of TEA molecules, resulting in increased hydrophobicity, and therefore, later elution compared to ssDNA. Increasing the temperature above the melting point (95 °C) resulted in only one cluster of signals around 11–12 min, which reflects multiple ssDNA species. Similarly to the profile observed at 75 °C, the large shift in elution time observed (from 18 to 11.5 min) corresponded to the change of the ssDNA conformation (lower hydrophobicity) rather than to the increase in temperature itself, which only had a minor influence. Additionally, the peak shape and resolution improved at higher column temperatures, as previously shown in the literature for small DNA fragment analysis.^39^ As a consequence, the broad peak observed at 55 °C resolved in multiple signals at 95 °C corresponding to different ssDNA species present in the rAAV sample.

**Figure 2:**
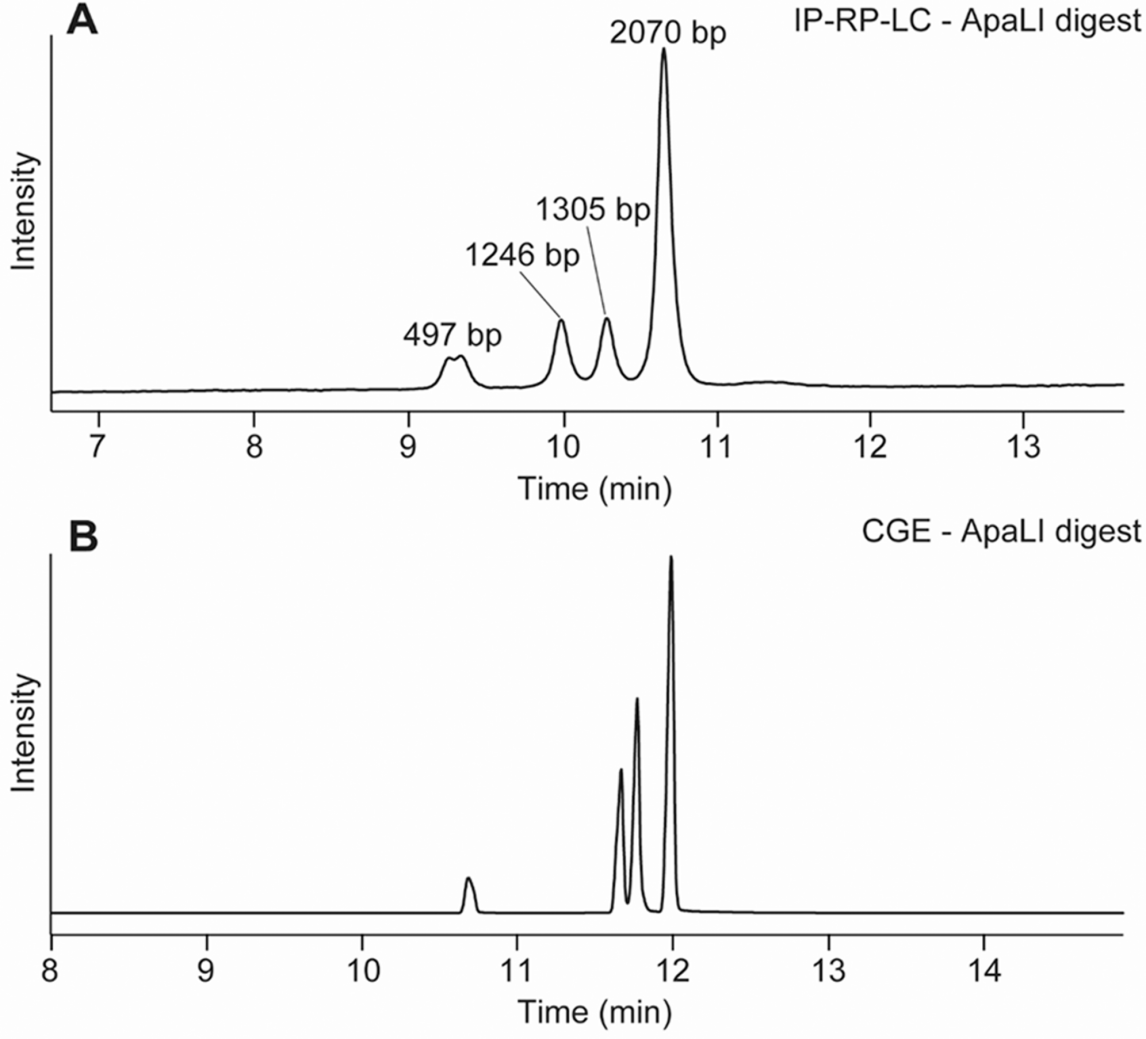
Analysis of the *Apa*LI plasmid digest by (A) IP-RP-LC and (B) CGE-LIF.

To further confirm that these signals arise from the genomic material, we analyzed the corresponding AAV8-V empty capsid sample (containing no DNA) from the same batch and performed an identical sample preparation. Figure S2 shows a comparison between AAV8-V full and empty samples using an analysis temperature of 95 °C. The AAV8-V empty sample showed only minor signals (zoom Figure S2), indicating that the detected peaks in the AAV8-V full sample come from the loaded genome. To get more information on the resolving power of the method, a sample consisting of fragments of different sizes was analyzed. To this end, a plasmid with a length of 5118 bp was digested using *Apa*LI, which resulted in four fragments with sizes of 2070 bp, 1305 bp, 1246 bp, and 497 bp. IP-RP-LC analysis showed four distinct signals separated by the length of DNA due to the ion pairing with TEA, resulting in a higher hydrophobicity the longer the DNA fragments are (Figure 2A). Under the applied conditions, a baseline separation of the two fragments, which differed only in 59 base pairs in size, could be achieved. To corroborate our results, we analyzed the same plasmid digest using a CGE-LIF approach published recently.^18,29^ CGE-LIF showed very similar profiles compared to IP-RP-LC with more efficient peak shapes for CGE-LIF, which is an intrinsic characteristic of this technique. In IP-RP-LC analysis, the peak at 497 bp showed a double peak. This peak was also broader in the CGE electropherogram (Figure 2B), also indicating a possible mixture of two DNA fragments such as a clipped variant or an unspecific cleavage. Additionally, while for the CGE-LIF method, an SYBR green dye, which binds ssDNA, is necessary, our IP-RP-LC method can directly detect DNA by UV. This opens the possibility that DNA can be measured at 95 °C, which is not possible with SYBR green due to the missing interaction between DNA and dye at these temperatures. Furthermore, LC has some benefits in the view of user-friendliness and reproducibility^36^ and opens the possibility of peak fractionation for further characterization (which may be challenging by CGE-LIF due to the low volumes employed). We also analyzed a digest of the same plasmid with *Sna*BI and *Xba*I, resulting in two fragments of 366 and 4752 bp (Figure S3). The 366 bp fragment eluted at around 9 min, while 4752 bp eluted much later (around 11.5 min) as expected. Overall, CGE and IP-RP-LC analysis resulted in very comparable results with higher peak efficiency for CGE. Finally, we analyzed one of the most complex AAV samples (AAV2-V) over the course of 3 days, resulting in a very similar profile and retention times for all three measurements on the 3 days with RSD values ≤ 4.7% for the AAV genome peak (11.2 min) (Figure S4).

### Discrimination between Internal and External DNA Using IP-RP-LC in Combination with Benzonase Treatment

During downstream processing, AAV samples are often treated with Benzonase in order to remove the potential DNA material present outside the capsid.^45^ Yet, in some cases, the remaining external DNA material can be observed in AAV preparations. Therefore, we evaluated if we could discriminate between internal and external DNA by combining our method with a Benzonase treatment prior to analysis.

**Figure 3:**
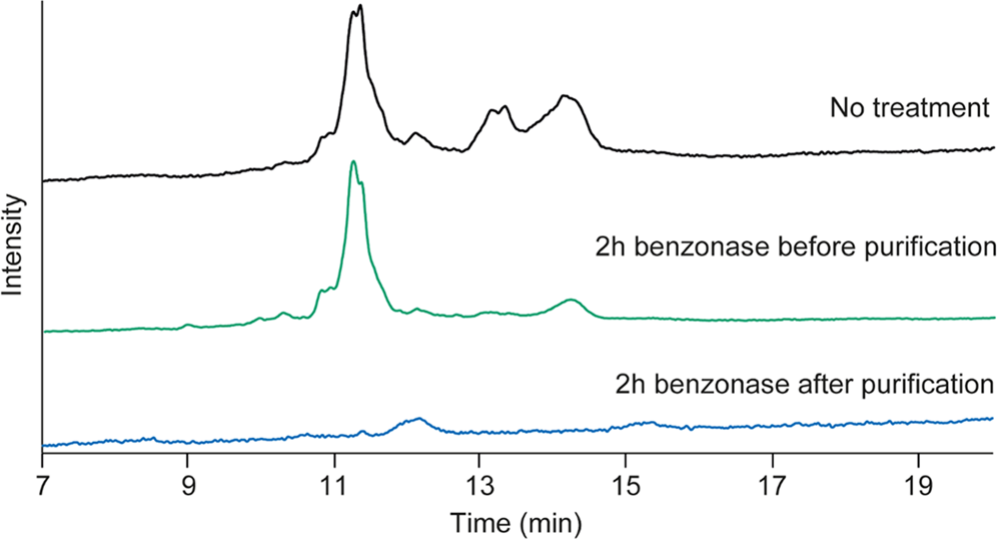
IP-RP-LC analysis of the AAV2-V genome at 95 °C without any Benzonase treatment (black trace), after 2 h Benzonase treatment before DNA purification (green line), and 2 h Benzonase treatment after DNA purification (blue trace).

As a test sample, we selected a rAAV, which showed a very complex IP-RP-LC profile with multiple signals, which were suspected to be external DNA (Figure 3). This sample was a rAAV from serotype 2 (AAV2-V), which should contain a genome of around 2.5 kb. After analysis, the expected genome was detected at around 11 min together with several additional larger DNA species (Figure 3, black trace). To investigate the location of these DNA impurities (external or internal), two Benzonase digestions were performed. One before and one after the disassembly of the capsids and subsequent DNA purification. After incubation for 2 h with Benzonase before capsid disassembly (Figure 3 green trace), a decrease in the intensity of larger-sized species was observed, in particular for the peaks between 12 and 14 min. These results suggest that these DNA species are indeed not packed into the AAV2-V particles and therefore digested, whereas the rAAV genome is resistant to Benzonase digestion due to its protection by the protein capsid. For the peak eluting at around 14.5 min, a significant decrease of signal was observed, yet a minor signal remained present. Due to its late elution, this signal should correspond to a DNA species with a size above 10 kb, which cannot be integrated into AAV. Therefore, we speculate that the remaining signal may arise from incomplete digestion or from other non-DNA impurities. AAV2-V was also incubated with Benzonase for 2 h after capsid disassembly and DNA purification (Figure 3 blue trace). After this procedure, no signal for the rAAV genome was observed. Only a minor signal, around 12 min, which was resistant to Benzonase digestion, was detected in the chromatogram. This signal was also observed in the AAV2-V empty sample. For this reason, we concluded that these peaks do not correspond to DNA but rather to minor protein impurities.

### IP-RP-LC for Integrity Assessment of Different-Sized AAV Genomes

AAVs can bear diverse genomes, and their genomic impurities can vary from sample to sample. Therefore, we purchased rAAV samples loaded with different-sized genomes and degrees of heterogeneity and applied the developed IP-RP-LC approach for their characterization. During method development, we observed an extraordinary complexity of rAAV samples, often leading to ambiguous results and difficult data interpretation. To increase comprehensiveness, we decided to flank our LC technique with CGE and SV-AUC. Since all techniques use very different separation principles, a more accurate picture of the genome integrity can be retrieved when combining them.

**Figure 4:**
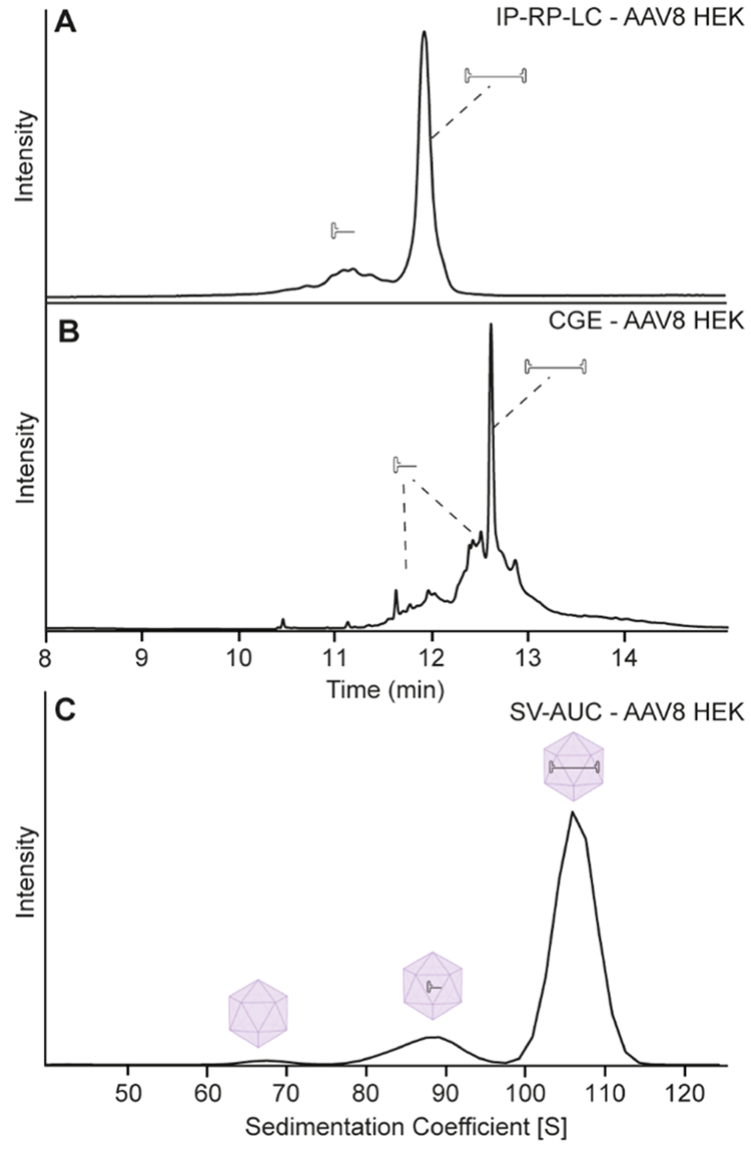
Analysis of AAV8-HEK after DNA purification by (A) IP-RP-LC and (B) CGE-LIF or (C) analysis of the intact rAAV particles with SV-AUC.

AAVs can accommodate up to 4.8 kbp of the genomic material. To evaluate the performance of the IP-RP-LC approach, we analyzed two AAV8 loaded with different length ssDNA. One of the selected AAV8 samples was expressed in HEK293 cells and was loaded with a 4.6 kbp genome. Figure 4A shows the IP-RP-LC separation obtained for AAV8-HEK with one main peak at 12 min representing the 4.6 kb ssDNA genome and some shorter DNA fragments eluting upfront. CGE-LIF showed a very similar profile with a higher number of smaller fragments separated from the main peak. In particular larger fragments migrating around 12.5 min were not resolved with IP-RP-LC (Figure 4B). The SV-AUC analysis perfectly matches the results obtained by the other two approaches, with one main peak at 105 S, as well as some particles with shorter DNA at 80–95 S (Figure 4C). In contrast to IP-RP-LC and CGE, SV-AUC is also capable of visualizing empty capsids at around 65 S.

**Figure 5:**
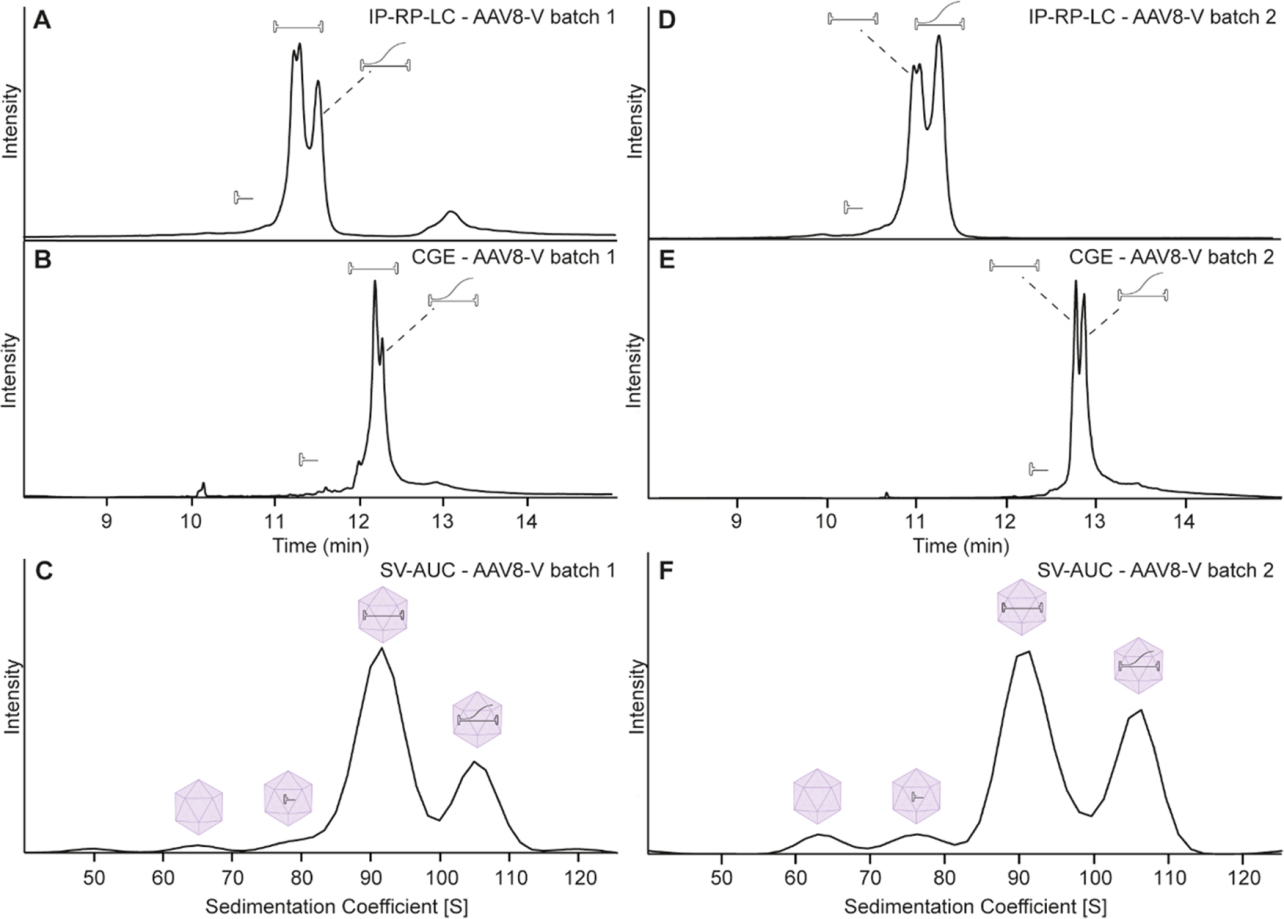
Analysis of AAV8-V batch 1 after DNA purification by (A) IP-RP-LC and (B) CGE-LIF or of the intact rAAV particles with (C) SV-AUC, as well as AAV8-V batch 2 by (D) IP-RP-LC, (E) CGE-LIF, and (F) SV-AUC.

For a shorter genome, we selected a rAAV8-V material produced in Sf9 insect cells and containing a theoretically loaded genome of approximately 2.5 kbp (AAV8-V Batch 1). Analysis of the AAV8-V batch 1 sample with IP-RP-LC showed a more complex profile comprising two distinct peaks, which were partially separated, as well as small signals eluting before and after the main peak (Figure 5A). A similar picture could also be observed with CGE-LIF, where two high abundance peaks were partially separated (Figure 5B). In IP-RP-LC, additionally, a partial resolution of the first high abundance peak was observed. We speculate that most probably they correlate to two ssDNA fragments with a very similar length but different hydrophobicity (e.g., sense and antisense), which were not resolved with CGE-LIF or SV-AUC, as their separation mechanisms are not affected by the hydrophobicity of the molecule. The species with faster mobility corresponds to the 2.5 kbp target DNA and the slower signal to a higher-sized species. SV-AUC further confirmed the findings as it also showed a very clear separation of two distinct species. Capsids containing 2.5 kbp DNA were detected around 90 S, and the heavier species were observed at 105 S (Figure 5C). The longer ssDNA construct most likely represents an elongation of the ssDNA genome with part of the plasmid backbone to the maximum loading capacity of a rAAV particle. Similar findings were also observed by another study using charge detection mass spectrometry as well as mass photometry.^46^ This effect might be due to the fact that shorter ssDNA has not had the optimal loading length for rAAV particles and therefore can result in unwanted elongation with the production plasmid backbone. Because this could lead to the transfer of some selection marker genes into the patient, these species should be very closely monitored and kept at a minimum to guarantee a safe rAAV product.

Next to the elongated genome, we detected some additional low abundance signals before the main peak corresponding to shorter DNA fragments. These low abundance signals are more clearly detectable by CGE-LIF. Also, in SV-AUC, capsids containing smaller DNA fragments were detected at 75–80 S. AAVs containing shorter DNA fragments are often observed in rAAV samples and have been previously reported.^28^ These rAAV capsids incorporate only a part of the expected genome, and they may lead to lower efficiency of the gene therapy product due to the lack of the target genome and can potentially be immunogenic and/or genotoxic for the patient, therefore requiring close monitoring. Finally, following the main peak, some signals were detected with CGE-LIF and IP-RP-LC. Due to the large size of these signals, we suspected that they correspond to external DNA, which was not completely removed by the Benzonase treatment during downstream processing, similar to the case of AAV2-V (Figure 3). Also, the fact that these signals are not detected by AUC suggests that they are not packed into the capsid; otherwise, a signal larger than 110 S would be expected.

In view of all of these observations, we also analyzed a second batch of AAV8 loaded with the 2.5 kbp genome (AAV8-V Batch 2). The IP-RP-LC results showed a similar picture; however, it seems that the larger DNA signal at 13 min is not present in this batch anymore. This again suggests that this large DNA was indeed external DNA from the production process, which was not properly removed in batch 1 (Figure 5D). Similarly, CGE-LIF also shows a slightly lower signal for the larger DNA species (Figure 5E), with the remaining ones most likely being dsDNA artifacts as previously reported by Hutanu et al.^18^ While the LC data suggest a lower amount of the elongated genome in batch 1, batch 2 shows nearly a 1:1 ratio. This finding was supported by CGE-LIF data as well as SV-AUC data (Figure 4F). Overall, these results demonstrate that the IP-RP-LC method is very well suited for batch comparability studies and thus adds an additional technique to the analytical toolbox of rAAVs.

**Figure 6:**
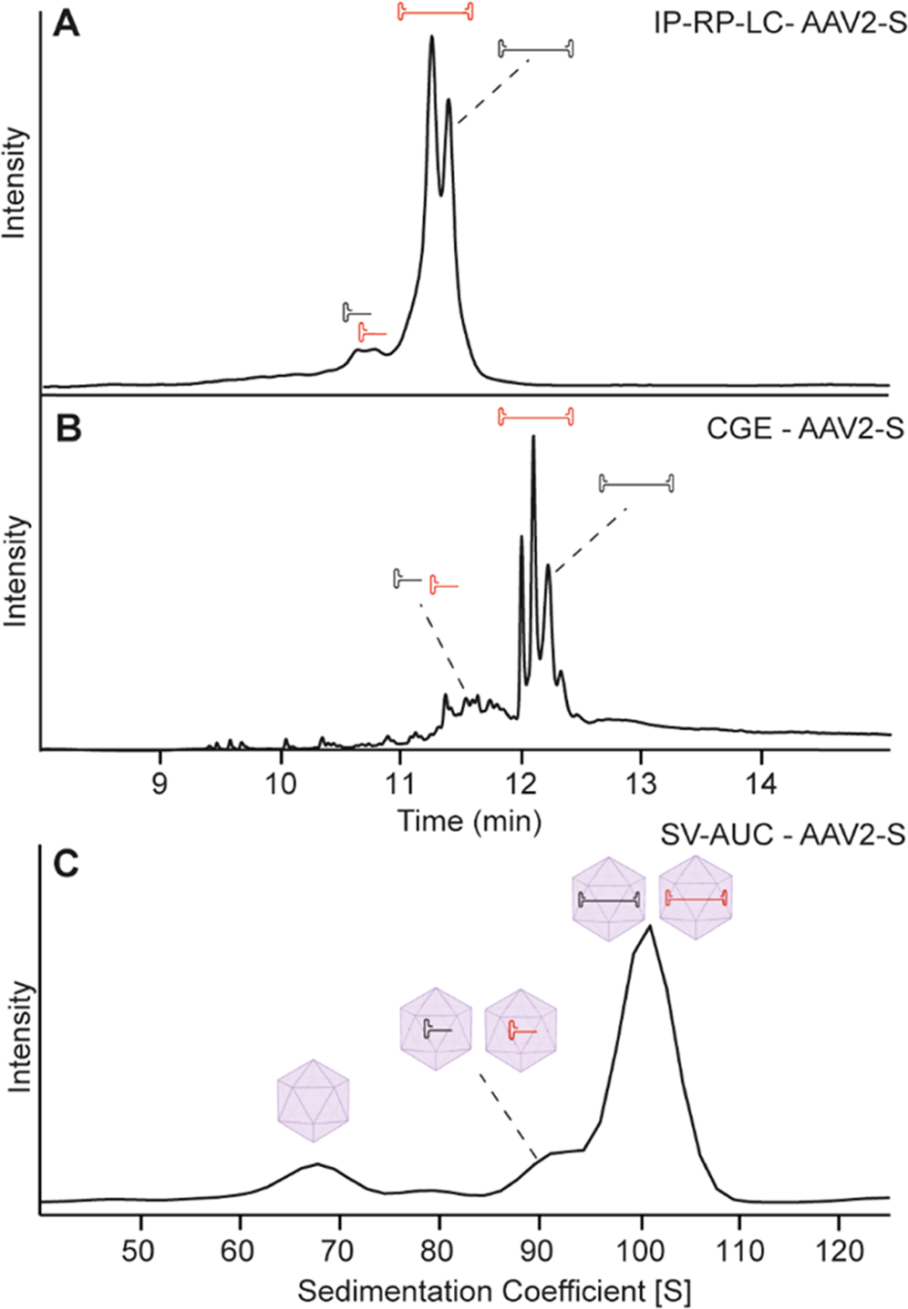
Analysis of AAV2-S after DNA purification by (A) IP-RP-LC and (B) CGE-LIF or of the intact rAAV particles with (C) SV-AUC.

As a last case, we applied the IP-RP-LC method to detect rAAV genomic contaminations. The AAV-2S sample (3.3 kbp) contained contamination by another AAV2 strain with a different genome (4.2 kbp, as indicated upfront by the supplier). The IP-RP-LC profile of the AAV-2S sample showed two clear main peaks and an additional small signal around 10.5 min (Figure 6A). The first peak represents the intended genome with a length of around 3.3 kbp, whereas the second represents the contaminant genome with a length of around 4.2 kbp. Furthermore, fragmented DNA was detected before the main peak, which was higher in intensity compared to the previously analyzed AAV8 samples. CGE-LIF largely supports these results by detecting two peaks, as well as small fragments, before the main peak (Figure 6B). Next to these two main peaks, an additional peak in the CGE profile at 12 min was observed, which was not detected by IP-RP-LC or SV-AUC. It cannot be excluded that this is a dsDNA or multiplex artifact due to incomplete denaturation or as a consequence of the very low concentration of this sample. Additional artifacts were observed throughout the complete analysis in CGE-LIF (Figure S5 marked with *), whereas in IP-RP-LC, no comparable signals were detected (only an increase in the baseline between 23 and 30 min due to the gradient). To exclude carryover effects as a cause for the unexpected signals, blank injections were performed before and after each CGE analysis. These injections were blank for two different analyses on two different devices, so we concluded that the cause for the observed signals must lie within the specimen (not shown). In numerous rAAV samples not mentioned in this study, we observed that CGE tends to have more artifacts in complex samples, in general. It should be mentioned that for this AAV2-S sample, the absolute intensity was slightly lower when compared to other rAAV analyses (most likely due to the lower virus titer), which could also play a role in the discrepancy between LC and CGE for this sample. Looking at the SV-AUC data, empty capsids were observed between 65 and 70 S, followed by some rAAV particles containing shorter DNA fragments between 75 and 95 S (Figure 6C). In contrast to IP-RP-LC and CGE-LIF, only one peak at 100 S was detected. These results illustrate the strength of SV-AUC to discriminate between different species and give a complete picture of the capsid filling state. However, smaller differences in genome length (only around 800 bp) may be challenging to detect by SV-AUC using standard conditions, illustrating once again the complementarity of these techniques.

## Conclusions

We have developed an IP-RP-LC approach for the analysis of the genomic material loaded in rAAV samples. We demonstrated the capacity of the approach to characterize the denatured ssDNA material from rAAV samples in a simple, fast, economic, and user-friendly way. In order to identify strengths and limitations, we compared our approach with the established techniques CGE-LIF^18,29^ and SV-AUC^18,20^ in a variety of rAAV samples. Results between all techniques were mostly consistent, which highlights the general applicability of the proposed method. Running the analysis at 95 °C allows to melt dsDNA and thereby analyze only ssDNA in contrast to the CGE-LIF approach, which also detects and separates dsDNA^18^ and other secondary structures.^29^ By using UV detection, we avoid the drawbacks of DNA dyes and have a direct detection of target DNA. Furthermore, LC is already established in the pharmaceutical industry for biopharmaceutical and small molecule drug characterization and therefore can be easily implemented. Comparison with CGE-LIF and SV-AUC supported our findings on the presence of elongated genomes and fragments of the genome of interest in a variety of rAAV samples. When the material is scarce, SV-AUC is not a viable option (approx. 400 μL consumption for a low viral titer), while IP-RP-LC requires only around 10–20 μL per injection, and CGE can perform multiple injections out of the same amount. IP-RP-LC was able to separate the genome of a rAAV2-S sample from a contaminant differing in 800 bp. Here, the IP-RP-LC and CGE-LIF approaches showed their benefit over the SV-AUC method, which was not able to resolve smaller differences in ssDNA under standard conditions. Overall, CGE-LIF provided higher peak efficiency compared to IP-RP-LC. On the other hand, IP-RP-LC allows peak fractionation and further characterization (e.g., by NGS) in a straightforward way. Furthermore, with some adaptations (e.g., the use of volatile ion pairing reagents or integration in 2D-LC with an MS-compatible second dimension), IP-RP-LC could be hyphenated with mass spectrometry for direct peak identification. Overall, the proposed IP-RP-LC is a very powerful alternative for the genome integrity assessment of rAAVs that can be easily adopted in QC labs, thus complementing the analytical toolbox of these next-generation therapeutics.

## Supporting Information Available

RP-LC analysis of VP proteins before and after purification; comparison of AAV8-V full and empty samples by IP-RP-LC; analysis of the plasmid digest by IP-RP-LC and CGE-LIF; analysis of AAV2-V in triplicates; IP-RP-LC chromatogram of AAV-2V and blank and comparison to CGE-LIF; and additional experimental information (PDF)
