# Supplementary material for: Reversed Phase-Liquid Chromatography for Recombinant AAV Genome Integrity Assessment
Source: Anal Chem. 2023 May 23;95(22):8478–86. doi: 10.1021/acs.analchem.3c00222 (PMC10248999; doi:10.1021/acs.analchem.3c00222)
Supplement: Supplementary file 1 — ac3c00222_si_001.pdf [file ac3c00222_si_001.pdf]

Supporting information

Reversed phase liquid chromatography for recombinant AAV genome integrity assessment

Christoph Gstöttner[a#], Andrei Hutanu[b#], Sacha Boon[a], Aurelia Raducanu[c], Klaus Richter[d], Markus Haindl[c], Raphael Ruppert[c*], Elena Dominguez-Vega[a*]

[a]Leiden University Medical Center, Center for Proteomics and Metabolomics, 2333ZA Leiden, The Netherlands.

[b]Pharma Technical Development Analytics, F. Hoffman-La Roche AG, 4070 Basel, Switzerland.

[c]Pharma Technical Operation Cell- and Gene Therapy, Roche Diagnostics GmbH, 82377 Penzberg, Germany.

[d]Coriolis Pharma Research GmbH, 82152 Planegg, Germany

C.G. and A.H. are shared first authors.

*R.R. and E.D.-V. are shared last authors.

Corresponding Author:
Elena Domínguez-Vega
e.dominguez_vega@lumc.nl

Table of Content

Supporting figure 1.. S2
Supporting figure 2.. S3
Supporting figure 3.. S4
Supporting figure 4.. S5
Supporting figure 5.. S6
Supporting information methods..S7-S8

Supporting information Figures

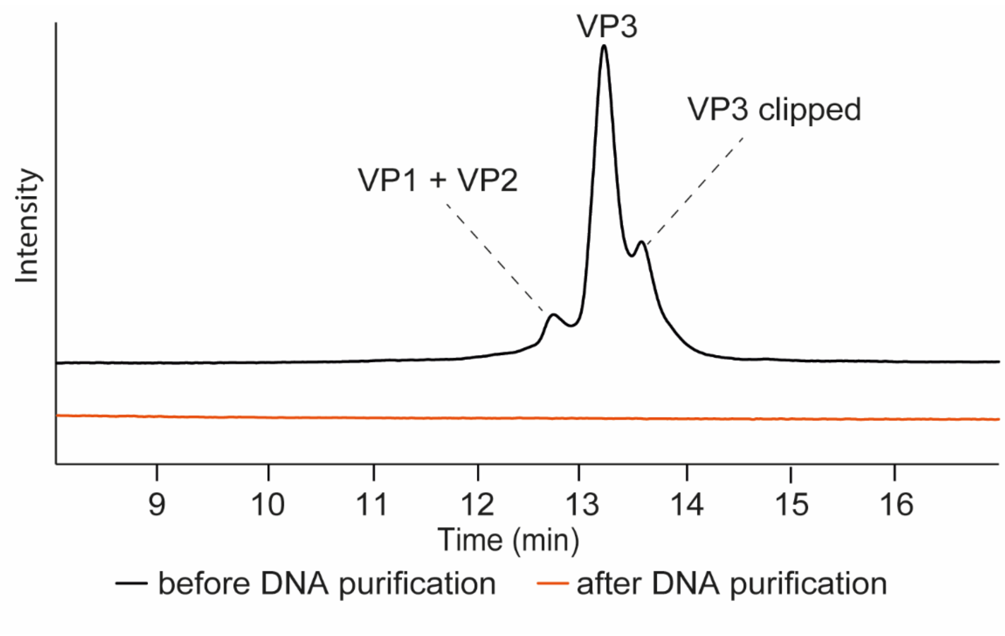

Figure S1: RP-LC analysis of a AAV8-V full sample before DNA purification (black line) and after DNA purification (orange line).

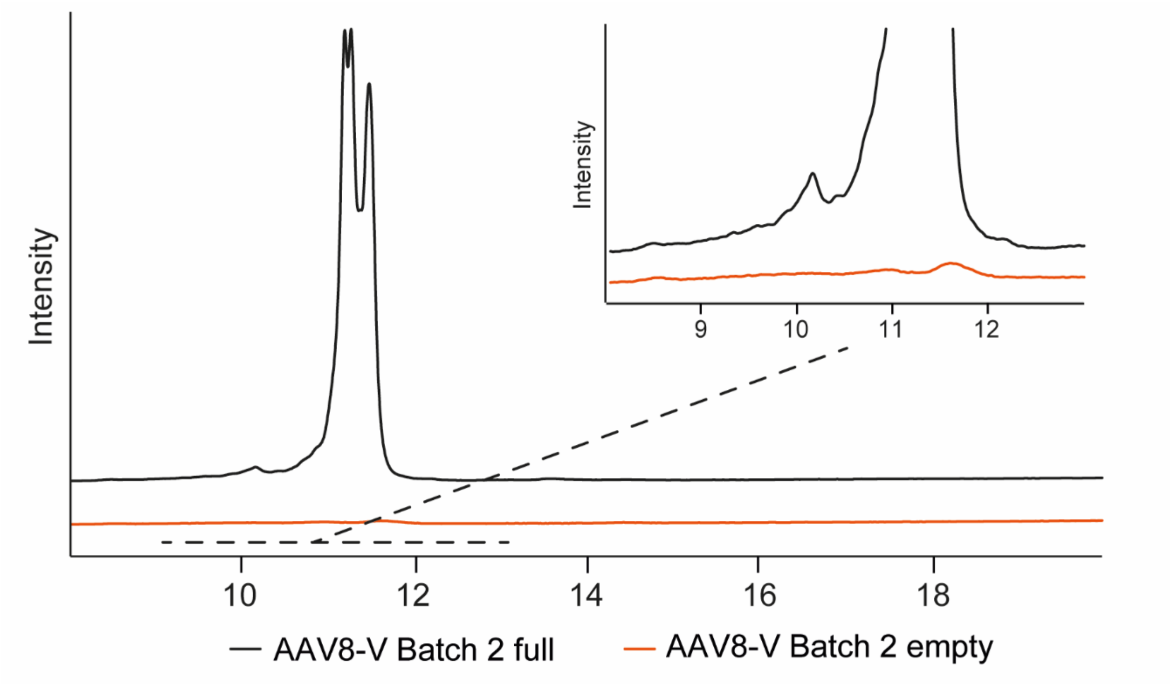

Figure S2: IP-RP-LC analysis of a AAV8-V full sample (black line) and a AAV8-V empty sample orange line. Additionally, a zoom in the elution region is depicted.

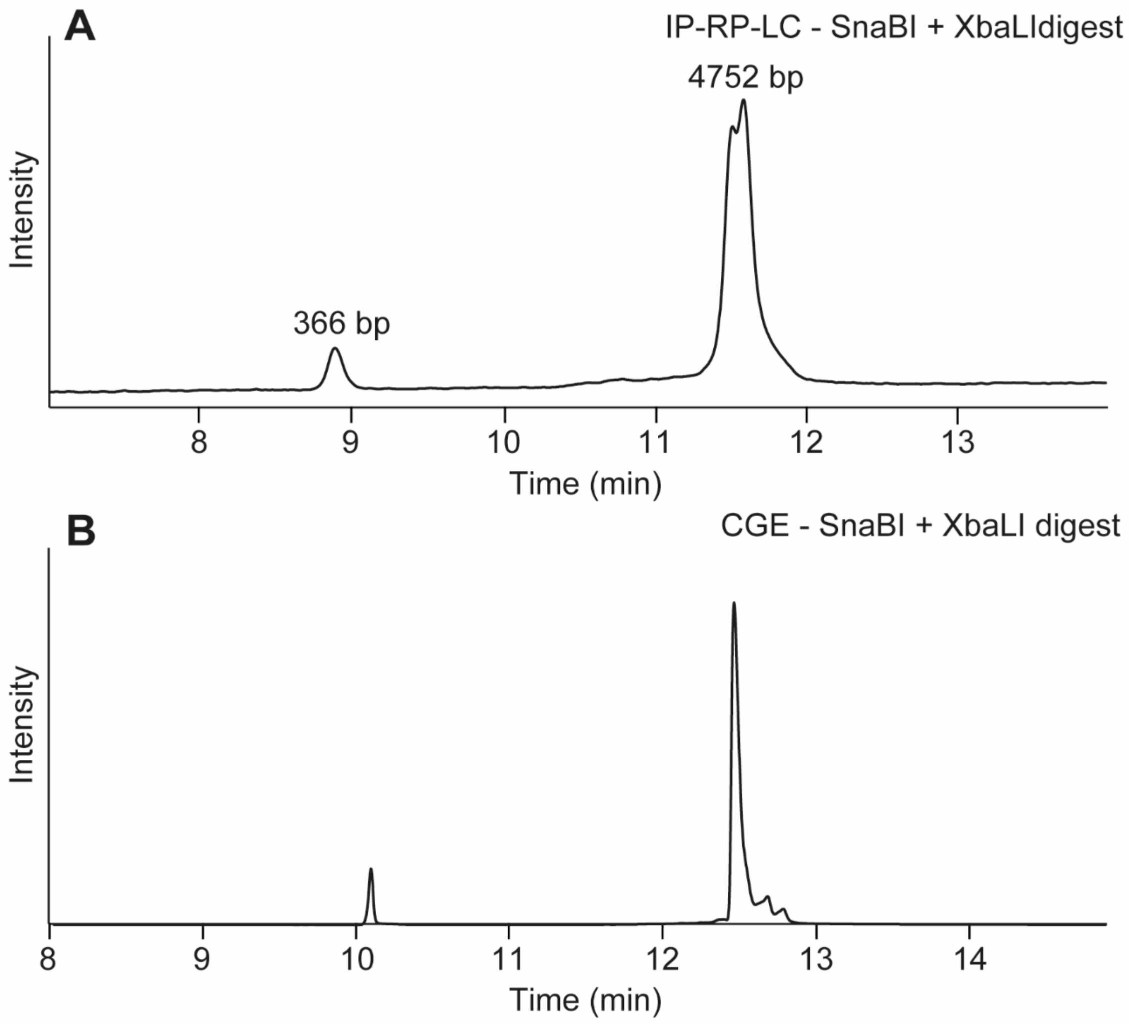

Figure S3: Analysis of a SnaBI + XbaLI plasmid digest by A) IP-RP-LC and B) CGE-LIF.

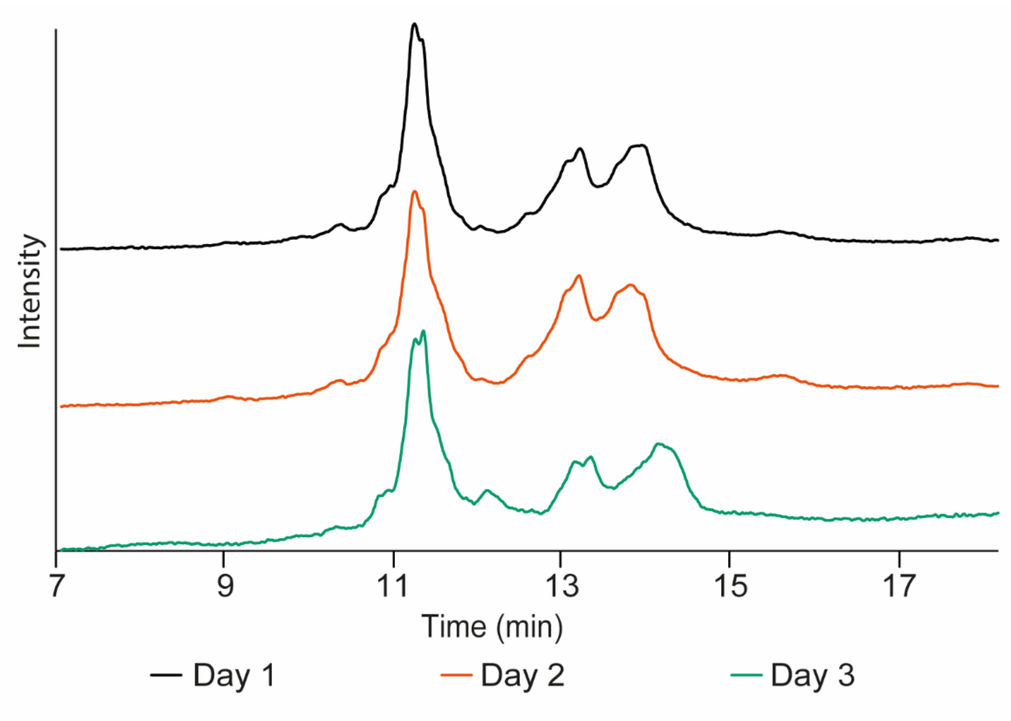

Figure S4: IP-RP-LC analysis of AAV2-V on three different days.

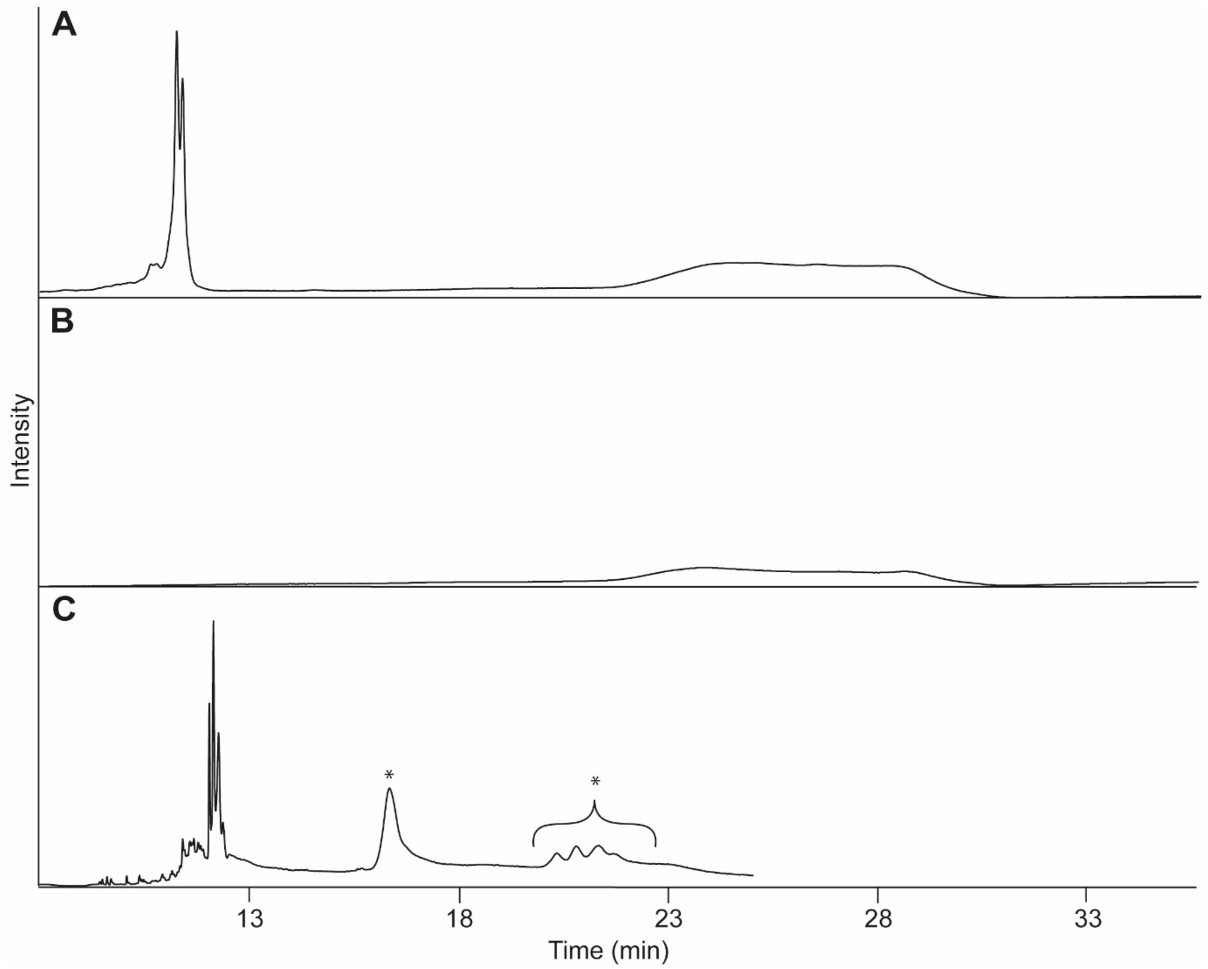

Figure S5: Analysis of A) AAV2-S by IP-RP-LC, B) Blank analysis by IP-RP-LC and AAV2-S by CGE-LIF detection. DNA artifacts are marked with a *.

Supporting information methods

Information S1 – Reagents and materials

Acetonitrile was provided by Actuall Chemicals (Oss, The Netherlands). For all mobile phases, demineralized water from an Elga water purification system was used. 1 M aqueous solution of triethylammonium acetate (TEAA) was purchased from Fluka (Buchs, Switzerland). The used SYBR™ Green II RNA Gel Stain 10,000 x concentrate in DMSO, UltraPure™ DNase/RNase-Free Distilled Water, FastRuler Middle Range DNA Ladder and the DNAPac RP column 2.1 x 100 mm with a particle size of 4 µm were obtained from Thermo Fisher Scientific (Waltham, Massachusetts). The BioResolve RP mAb Polyphenyl column 2.1 x 150 mm with a particle size of 2.7 µm was purchased from Waters (Milford, Massachusetts). The QIAquick PCR purification kit was purchased from Qiagen (Hilden, Germany). Benzonase Nuclease, $MgCl_2$ and $CaCl_2$ dihydrate were from Sigma Aldrich (St. Louis, Missouri). Phosphate buffered saline (PBS) tablets (1 tablet in 1L water results in 140 mM NaCl, 10 mM phosphate buffer, 3 mM KCl, pH 7.4), glacial acetic acid, Polyvinylpyrrolidone (PVP), 10x tris(hydroxymethyl)aminomethane (Tris) borate EDTA (TBE) buffer, Tween 20, difluoroacetic acid (DFA), Hellmanex 3 and Urea were supplied by Sigma-Aldrich/Merck KGaA (Darmstadt; Germany). Tris-HCl was obtained from Invitrogen (Waltham, Massachusetts). Bare fused silica CE capillaries with an inner diameter of 50 µm were purchased from Polymicro Technologies /Molex LLC (Phoenix, USA). Plasmid DNA (Cat. no. VB190926-1395dab) was provided by VectorBuilder Inc. (Chicago, USA). ApaLI, SnaBI and XbaI restriction endonucleases were obtained from New England Biolabs (Ipswich, USA). 10x PBS (1.55 M NaCl, 27 mM sodium phosphate dibasic, 15 mM KCl, pH 7.2) and 10% Pluronic F68 were obtained from Gibco Thermo Fisher Scientific (Waltham, Massachusetts). Ethanol absolute was purchased from VWR (Darmstadt, Germany). Fill hole gaskets (neoprene) and fill hole screws were provided by Spin Analytical (South Berwick, Maine).

Information S2 - Analysis of AAV proteins with RP-LC

For the analysis of rAAV proteins the AAV sample was either disassembled using 10% acetic acid in the case of direct analysis or purified with the QIAquick PCR purification kit and thereby disassembled in the using the provided binding buffer (Buffer PB). For the analysis an Agilent 1200 series instrument equipped with a quaternary pump (G1311A) combined with a degasser (G1322A), autosampler (G1367D) with thermostat (G1330B), column oven (1316B) and variable wavelength detector (G1314C) with a standard cell was used (Agilent Technologies, Waldbronn, Germany) was employed. Analysis rAAV proteins either before or after purification was performed using a BioResolve RP mAb Polyphenyl column 2.1 x 150 mm column at 80 °C with 0.1% DFA in water as mobile phase A and 0.1% DFA in ACN as mobile phase B. The starting condition was 35% B which was gradually increased to 50% B in 25 min followed by an increase to 95% B in 1 min. After 4 min cleaning of the column at 95% B the starting conditions were reached during a 1 min gradient followed by a re-equilibration of the column for 9 min resulting in a total analysis time of 40 min with a constant flowrate of 0.15 mL/min. The injection volume was 10 µL. The proteins were detected using UV detection at 280 nm.

Information S3 - Analysis of rAAV by sedimentation-velocity analytical ultracentrifugation (SV-AUC)

For measurements of sedimentation velocity an Optima analytical ultracentrifuge from Beckman-Coulter (Brea, California) with an 8-hole AN-50 Ti analytical rotor and 12-mm charcoal-epon double-

sector centerpieces was used. Cleaning upfront AUC measurements was performed using AUC cell washers (Spin Analytical, South Berwick, Maine), and was confirmed by two consecutive absorbance intensity scans through empty cells. Afterwards the sample sector was filled with 380 µL rAAV sample and the reference sector with 400 µL formulation buffer. The formulation buffer consisted of 1 x PBS (Gibco) and 0.001% Pluronic F-68 in case of AAV 8 samples or 1 x PBS (Gibco) with 0.014% Tween 20 in case of AAV2 samples. After a temperature equilibration of sample and rotor with resting rotor, the sedimentation profiles were recorded using the absorbance detection optics in intensity mode (at 230 nm and 280 nm) at 12000 rpm. Scans 1-50 were analyzed using UltraScan III using a partial specific volume of 0.73 mL/g and a radial data range from approximately 0.03 cm above meniscus position to 7.10 cm. The raw data were pre-processed (meniscus position fit, time invariant and radial invariant noise corrections) by using 2-dimensional spectrum analysis (2DSA). Afterwards, the obtained results were refined by using parametrically constrained spectrum analysis (PCSA) applying the straight line (SL) model. Estimation of confidence intervals was performed by 100 Monte Carlo (MC) iterations on top of the PCSA-SL results. The calculations were carried out at Coriolis Pharma Research GmbH on an in-house Boston Server cluster with 128-core AMD Epyc™ 7702 processors. Pseudo-3D distributions were analyzed with a sample-specific s-value range bin set to determine the content of empty capsids, full capsids, light molecular weight species (LMWS) and heavy molecular weight species (HMWS).
